# Association of early mobility with the incidence of deep-vein thrombosis and mortality among critically ill patients: a post hoc analysis of PREVENT trial

**DOI:** 10.1186/s13054-023-04333-9

**Published:** 2023-03-03

**Authors:** Hasan M. Al-Dorzi, Samah AlQahtani, Abdulaziz Al-Dawood, Fahad M. Al-Hameed, Karen E. A. Burns, Sangeeta Mehta, Jesna Jose, Sami J. Alsolamy, Sheryl Ann I. Abdukahil, Lara Y. Afesh, Mohammed S. Alshahrani, Yasser Mandourah, Ghaleb A. Almekhlafi, Mohammed Almaani, Ali Al Bshabshe, Simon Finfer, Zia Arshad, Imran Khalid, Yatin Mehta, Atul Gaur, Hassan Hawa, Hergen Buscher, Hani Lababidi, Abdulsalam Al Aithan, Yaseen M. Arabi, for the Saudi Critical Care Trials Group

**Affiliations:** 1 Intensive Care Department, King Abdulaziz Medical City, Ministry of National Guard Health Affairs, Riyadh, Kingdom of Saudi Arabia; 2 King Abdullah International Medical Research Center, Riyadh, Kingdom of Saudi Arabia; 3 College of Medicine, King Saud Bin Abdulaziz University for Health Sciences, Riyadh, Kingdom of Saudi Arabia; 4 Intensive Care Department, King Abdulaziz Medical City, Ministry of National Guard Health Affairs, Jeddah, Kingdom of Saudi Arabia; 5 King Abdullah International Medical Research Center, Jeddah, Kingdom of Saudi Arabia; 6 King Saud Bin Abdulaziz University for Health Sciences, Jeddah, Kingdom of Saudi Arabia; 7 Interdepartmental Division of Critical Care Medicine, University of Toronto, Toronto, Canada; 8 Unity Health Toronto - St Michael’s Hospital, Toronto, Canada; 9 Li Ka Shing Knowledge Institute, Toronto, Canada; 10 Department of Medicine, University of Toronto, Toronto, Canada; 11 Medical Surgical ICU, Sinai Health, Toronto, Canada; 12 Department of Biostatistics and Bioinformatics, King Abdullah International Medical Research Center, Riyadh, Kingdom of Saudi Arabia; 13 Department of Emergency and Critical Care Medicine, College of Medicine, King Fahd Hospital of the University, Imam Abdulrahman Bin Faisal University, Dammam, Kingdom of Saudi Arabia; 14 Military Medical Services, Ministry of Defense, Riyadh, Kingdom of Saudi Arabia; 15 Department of Intensive Care Services, Prince Sultan Military Medical City, Riyadh, Kingdom of Saudi Arabia; 16 Department of Pulmonary and Critical Care Medicine, King Fahad Medical City, Riyadh, Kingdom of Saudi Arabia; 17 Department of Critical Care Medicine, King Khalid University, Asir Central Hospital, Abha, Kingdom of Saudi Arabia; 18 The George Institute for Global Health, University of New South Wales, Sydney, Australia; 19 Department of Anesthesiology and Critical Care, King George’s Medical University, Lucknow, India; 20 Critical Care Section, Department of Medicine, King Faisal Specialist Hospital and Research Center, Jeddah, Kingdom of Saudi Arabia; 21 Institute of Critical Care and Anaesthesiology, Medanta - The Medicity, Gurgaon, Haryana, India; 22 Intensive Care Department, Gosford Hospital, Gosford, NSW, Australia; 23 Critical Care Medicine Department, King Faisal Specialist Hospital and Research Center, Riyadh, Kingdom of Saudi Arabia; 24 Department of Intensive Care Medicine, Center for Applied Medical Research, St. Vincent’s Hospital, University of New South Wales, Sydney, Australia; 25 Intensive Care Division, Department of Medicine, King Abdulaziz Hospital, Ministry of National Guard Health Affairs, Al Ahsa, Kingdom of Saudi Arabia; 26 King Abdullah International Medical Research Center , Al Ahsa, Kingdom of Saudi Arabia

**Keywords:** Critical care, Rehabilitation, Outcomes, Mobility, Venous thromboembolism, Deep-vein thrombosis

## Abstract

**Background:**

This study assessed the mobility levels among critically ill patients and the association of early mobility with incident proximal lower-limb deep-vein thrombosis and 90-day mortality.

**Methods:**

This was a post hoc analysis of the multicenter PREVENT trial, which evaluated adjunctive intermittent pneumatic compression in critically ill patients receiving pharmacologic thromboprophylaxis with an expected ICU stay ≥ 72 h and found no effect on the primary outcome of incident proximal lower-limb deep-vein thrombosis. Mobility levels were documented daily up to day 28 in the ICU using a tool with an 8-point ordinal scale. We categorized patients according to mobility levels within the first 3 ICU days into three groups: early mobility level 4–7 (at least active standing), 1–3 (passive transfer from bed to chair or active sitting), and 0 (passive range of motion). We evaluated the association of early mobility and incident lower-limb deep-vein thrombosis and 90-day mortality by Cox proportional models adjusting for randomization and other co-variables.

**Results:**

Of 1708 patients, only 85 (5.0%) had early mobility level 4–7 and 356 (20.8%) level 1–3, while 1267 (74.2%) had early mobility level 0. Patients with early mobility levels 4–7 and 1–3 had less illness severity, femoral central venous catheters, and organ support compared to patients with mobility level 0. Incident proximal lower-limb deep-vein thrombosis occurred in 1/85 (1.3%) patients in the early mobility 4–7 group, 7/348 (2.0%) patients in mobility 1–3 group, and 50/1230 (4.1%) patients in mobility 0 group. Compared with early mobility group 0, mobility groups 4–7 and 1–3 were not associated with differences in incident proximal lower-limb deep-vein thrombosis (adjusted hazard ratio [aHR] 1.19, 95% confidence interval [CI] 0.16, 8.90; *p* = 0.87 and 0.91, 95% CI 0.39, 2.12; *p* = 0.83, respectively). However, early mobility groups 4–7 and 1–3 had lower 90-day mortality (aHR 0.47, 95% CI 0.22, 1.01; *p* = 0.052, and 0.43, 95% CI 0.30, 0.62; *p* < 0.0001, respectively).

**Conclusions:**

Only a small proportion of critically ill patients with an expected ICU stay ≥ 72 h were mobilized early. Early mobility was associated with reduced mortality, but not with different incidence of deep-vein thrombosis. This association does not establish causality, and randomized controlled trials are required to assess whether and to what extent this association is modifiable.

***Trial registration*:**

The PREVENT trial is registered at ClinicalTrials.gov, ID: NCT02040103 (registered on 3 November 2013) and Current controlled trials, ID: ISRCTN44653506 (registered on 30 October 2013).

**Supplementary Information:**

The online version contains supplementary material available at 10.1186/s13054-023-04333-9.

## Introduction

Immobility in patients admitted in the intensive care unit (ICU) is common due to the nature of critical illness and the interventions provided for its management, including sedatives, narcotics, and/or paralytic agents. Immobility increases the risk of multiple adversities that include muscle atrophy and weakness [1], insulin resistance, pressure injury [2], pneumonia [3], and venous thromboembolism (VTE) [4]. These adversities may be induced by multiple mechanisms such as reduced muscle loading and enhanced overproduction of reactive oxygen and nitrogen species [5], and may persist for several months and years after ICU discharge [6, 7]. Early mobility has been recommended for ICU patients [8], due to improvement in various outcomes [9–16]. A systematic review of 8 randomized controlled trials and 10 observational studies that evaluated the association between mobility with or without concomitant thromboprophylaxis and VTE in adult hospitalized patients observed variable mobility interventions across studies, and found mixed results on the association of mobility and VTE [17]. The heterogeneity of studies and the variable definition of mobility made it impossible to quantify any therapeutic ambulation dose [17]. The review concluded that mobility alone should not be considered as an adequate modality for thromboprophylaxis [17]. Another systematic review and meta-analysis of 39 randomized controlled trials, most had significant risks of bias, found that early mobility in critically ill patients was associated with lower incidence of deep-vein thrombosis (7 studies, 730 patients, risk ratio 0.16; 95% confidence interval (CI) 0.06, 0.47; *I*^2^ 0%), but not with mortality [18].

The role of early mobility for VTE prevention is not well studied. This study aimed to assess the association of early mobility with the incidence of proximal lower-limb deep-vein thrombosis and 90-day mortality of critically ill patients.

## Methods

### Setting

This was a post hoc analysis of the Pneumatic Compression for Preventing Venous Thromboembolism (PREVENT) trial (NCT02040103, ISRCTN44653506), a multicenter randomized controlled trial that was conducted in 20 sites in Saudi Arabia, Canada, Australia, and India. The Institutional Review Boards of participating centers approved the trial. Informed consent was obtained from all enrolled patients or their surrogate decision-makers. In this trial, adult medical, surgical, or trauma critically ill patients receiving pharmacologic thromboprophylaxis with an expected ICU stay of > 72 h were randomized to receive adjunctive intermittent pneumatic compression with pharmacologic thromboprophylaxis or pharmacologic thromboprophylaxis alone. Twice-weekly lower-limb ultrasonography was performed until ICU discharge, death, full mobility, or 28 days after enrollment, whichever occurred first. The trial found no effect on the primary outcome (incident proximal lower-limb deep-vein thrombosis) [19].

### Patients

The PREVENT trial enrolled 2003 patients. Except for the study intervention and the required periodic screening with lower-limb ultrasound, routine ICU management, including mobility, was as per the local standard of the participating centers. Mobility levels were documented daily up to day 28 in the ICU using the tool of the Agency for Healthcare Research and Quality Safety Program for Mechanically Ventilated Patients [20]. The tool includes an 8-point ordinal scale ranging from 0 (passively rolled or exercised by staff) to 7 (walking away from the bed/chair by at least four steps) (Additional file 1: Table S1) [20]. Mobility data were added to the data collection after 6 months of starting the trial and were documented on 1708/2003 (85.3%) of enrolled patients; these patients were included in the current analysis. We categorized patients based on the highest level of mobility in the first 3 calendar days into three groups: early mobility level 4–7 (at least active standing), early mobility level 1–3 (passive transfer from bed to chair or active sitting), and early mobility level 0 (passive range of motion).

### Data collection

For this study, we surveyed the participating centers for their mobility practices during the trial period (Additional file 1: Table S2). We extracted the following data for patients in the three mobility groups: demographic information, Acute Physiology And Chronic Health Evaluation (APACHE) II score, VTE risk factors before ICU admission (hospitalization in the preceding 3 months for any reason, paralysis or immobilization of a lower or upper extremity related to stroke or injury prior to hospital admission, active malignancy, recent surgery, acute stroke, trauma, personal history of VTE, family history of VTE, known thrombophilia, post-partum state within 3 months, and estrogen therapy), baseline (at enrolment) organ support of mechanical ventilation, vasopressors, renal replacement therapy, and randomization to adjunctive pneumatic compression versus control.

We also noted the study interventions and cointerventions during the ICU stay including the number of patients receiving pneumatic compression, graduated compression devices, prophylactic unfractionated heparin, prophylactic low molecular weight heparin, and antiplatelet therapy (aspirin or clopidogrel), the use of organ support (vasopressors, mechanical ventilation, renal replacement therapy, and presence of femoral central venous catheters.

The primary outcomes were incident proximal lower-limb deep-vein thrombosis, defined as new thrombosis detected by twice-weekly lower-limb ultrasonography after the third calendar day since randomization until ICU discharge, death, attainment of full mobility, or trial day 28, whichever occurred first, and 90-day mortality. Deep-vein thrombosis detected on trial days 1 to 3 was considered prevalent. Secondary outcomes included acute pulmonary embolism, ICU and hospital mortality, mortality at 28 days, duration of vasopressor use, vasopressor-free days, mechanical ventilation duration, ventilator-free days, ICU-free days, and length of stay in the ICU and hospital.

### Statistical analysis

We presented continuous variables as median with interquartile range and compared them using Mann–Whitney U test or Student t-test for the two-group comparisons (early mobility group 4–7 versus 0 and early mobility group 1–3 versus 0), depending on normality of distribution. We presented categorical variables as frequencies with percentages and compared them using the chi-square test or Fisher’s exact test, as appropriate.

We assessed the association of mobility level with categorical outcomes (incident proximal lower-limb deep-vein thrombosis and 90-day mortality) using Cox proportional models and with continuous outcomes (duration of vasopressor use, vasopressor-free days, mechanical ventilation duration, ventilator-free days, ICU-free days, and length of stay in the ICU and hospital) using generalized linear mixed models adjusting for the following covariables: randomization group (adjunctive pneumatic compression with pharmacologic thromboprophylaxis versus pharmacologic thromboprophylaxis alone), type of heparin (unfractionated heparin versus low molecular weight heparin), femoral central venous catheters, mechanical ventilation, vasopressor therapy, APACHE II score, and body mass index. The results were presented as adjusted hazard ratios (aHR) or beta estimates with 95% CIs. Kaplan–Meier survival curves were plotted to compare deep-vein-thrombosis-free time and survival time between the different mobility groups. Log-rank *p v*alues were computed. A *p* value < 0.05 was considered statistically significant. All analyses were conducted using SAS software, version 9.4 (SAS Institute, Cary, NC, USA).

## Results

The mobility practices of the participating centers during the trial periods are presented in Additional file 1: Table S2. Most centers had a protocol for early mobility during the trial with a physiotherapist providing treatments to a median of 10 patients on a median of 5 days per week.

### Characteristics of patients

Of the 1708 patients who were included in the analysis, only 85 patients (5.0%) had an early mobility level of 4–7 during the first 72 h and 356 patients (20.8%) had an early mobility level of 1–3 while 1267 patients (74.2%) had a mobility level of 0. The baseline characteristics of these patients are shown in Table 1. Patients with early mobility level 4–7 had lower APACHE II scores, received less organ support (vasopressor therapy, mechanical ventilation) and had slightly lower prevalence of femoral central venous catheters at baseline. Early mobility level varied between the countries (Saudi Arabia, Canada, Australia, and India) of the study patients** (**Table 1 and Additional file 1: Table S3). Additional data regarding source of admission, VTE risk factors and pertinent laboratory findings are shown in Additional file 1: Table S4.

**Table 1:** Baseline characteristics of patients who had early mobility levels 4–7, 1–3, and 0

	Early mobility 4–7 *N* = 85	Early mobility 1–3 *N* = 356	Early mobility 0 *N* = 1267	*p* value mobility 4–7 versus 0	*p* value mobility 1–3 versus 0
Age (years)—mean ± SD	56.6 ± 17.7	52.4 ± 20.0	59.1 ± 20.6	0.13	< 0.0001
Male sex—no. (%)	58 (68.2)	213 (59.8)	724 (57.1)	0.045	0.36
BMI (kg/m^2^)—median (IQR)	27.7 (24.5, 31.7)	26.3 (22.3, 30.5)	27.3 (23.4, 32.5)	0.58	0.002
Admission category—no. (%)					
Medical	66 (77.6)	275 (77.2)	971 (76.6)	0.18	0.96
Surgical	16 (18.8)	50 (14.0)	186 (14.7)		
Trauma	3 (3.5)	31 (8.7)	110 (8.7)		
APACHE II—mean ± SD	16.9 ± 7.5	18.5 ± 7.8	20.5 ± 7.8	< 0.0001	< 0.0001
Chronic health illnesses—no. (%)					
None	45 (52.9)	169 (47.5)	643 (50.7)	0.70	0.27
Chronic respiratory disease	19 (22.4)	93 (26.1)	229 (18.1)		
Chronic cardiovascular disease	7 (8.2)	62 (17.4)	199 (15.7)		
Chronic renal disease	6 (7.1)	26 (7.3)	173 (13.7)		
Chronic liver disease	2 (2.4)	3 (0.8)	35 (2.8)		
Immunosuppression	14 (16.5)	41 (11.5)	141 (11.1)		
Pre-ICU VTE risk factors—no. (%)					
None	53 (62.4)	185 (52.0)	491 (38.8)	< 0.0001	< 0.0001
Hospitalization in the past 3 months for any reason (excluding this hospital admission)	15 (17.6)	60 (16.9)	291 (23.0)		
Active malignancy (treatment within past 6 months or palliation)	5 (5.9)	30 (8.4)	127 (10.0)		
Recent surgery (in the last 48 h)	12 (14.1)	26 (7.3)	123 (9.7)		
Trauma	4 (4.7)	30 (8.4)	111 (8.8)		
Organ support at baseline—no. (%)					
Mechanical ventilation	30 (35.3)	138 (38.8)	930 (73.4)	< 0.0001	< 0.0001
Vasopressors	19 (22.4)	72 (20.2)	503 (39.7)	0.0015	< 0.0001
Femoral central venous catheter at baseline—no. (%)	7 (8.2)	199 (15.7)	44 (12.4)	0.06	0.12
Pharmacologic prophylaxis at baseline—no. (%)					
Unfractionated heparin	49 (57.6)	165 (46.3)	758 (59.8)	0.69	< 0.0001
Low molecular weight heparin	36 (42.4)	191 (53.7)	509 (40.2)		
Trial randomization—no. (%)					
Intermittent pneumatic compression	46 (54.1)	186 (52.2)	618 (48.8)	0.34	025
Control	39 (45.9)	170 (47.8)	649 (51.2)		
Country—no. (%)					
Australia	31 (36.5)	8 (2.2)	26 (2.1)	< 0.0001	< 0.0001
Canada	17 (20.0)	43 (12.1)	87 (6.9)		
India	9 (10.6)	87 (24.4)	86 (6.8)		
Saudi Arabia	28 (32.9)	218 (61.2)	1068 (84.3)		

Continuous variables were not normally distributed and were compared using Mann–Whitney *U* test. Categorical variables were compared using the chi-square test

SD, standard deviation; BMI, body mass index; APACHE, Acute Physiology and Chronic Health Evaluation; INR, international normalized ratio; PTT, partial thromboplastin time; VTE, venous thromboembolism; IQR, interquartile range

**Fig. 1:**
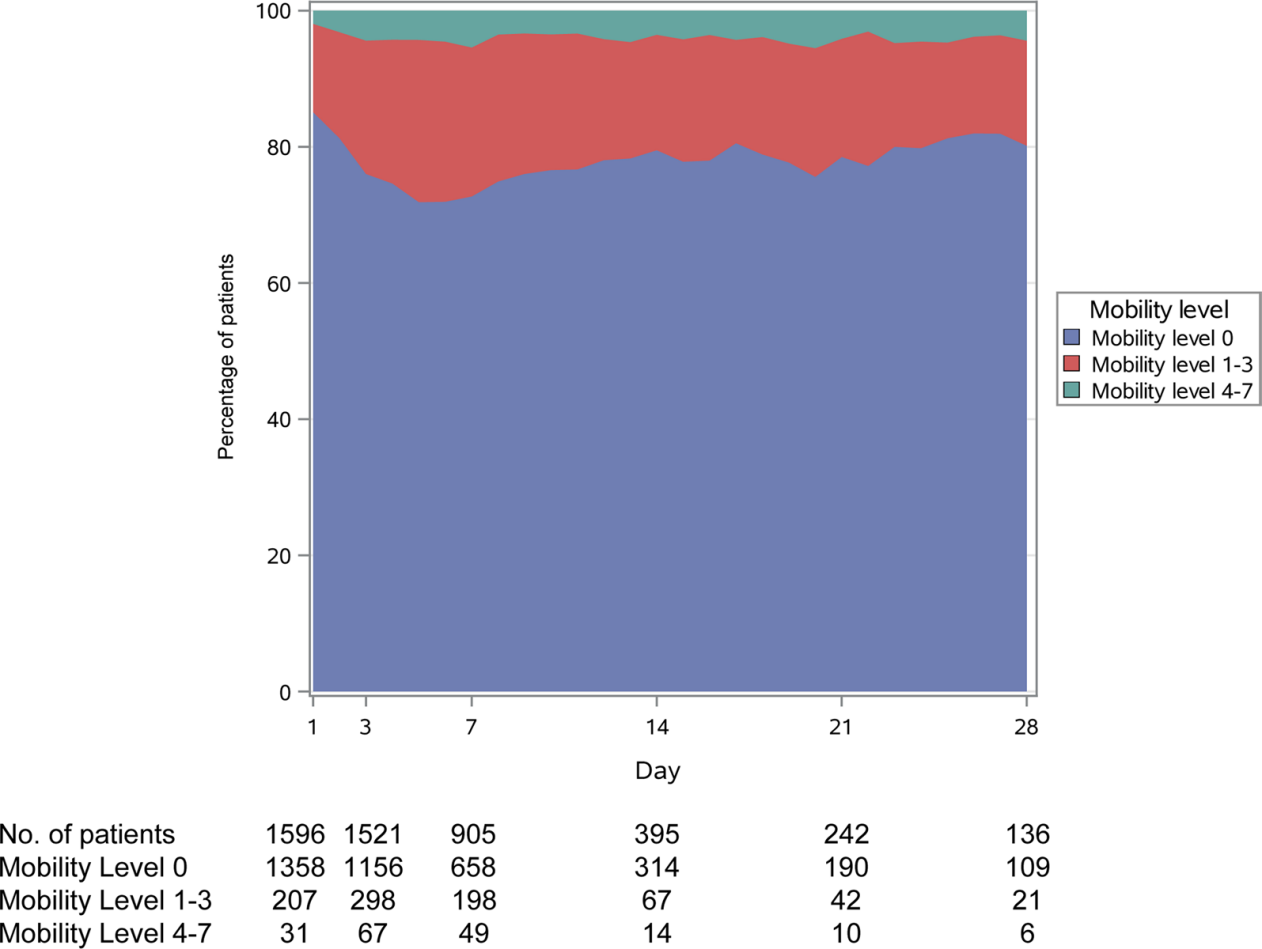
Daily mobility levels of the study cohort while in the intensive care unit up to 28 days. The change in mobility level over time was statistically significant (*p* < 0.0001)

During ICU stay, 247/905 (27.3%) patients had mobility level above 0 on day 7, 81/395 (20.5%) patients on day 14, 52/242 (21.5%) patients on day 21, and 30/139 (21.6%) patients on day 28 (Fig. 1). The change in mobility level over time was significant (*p* < 0.0001). Table 2 describes the treatments provided during ICU stay. There were no between-group differences in the use of adjunctive pneumatic compression.

**Table 2:** Interventions and co-interventions of patients with early mobility levels 4–7, 1–3 and 0

	Early mobility 4–7 *N* = 85	Early mobility 1–3 *N* = 356	Early mobility 0 *N* = 1267	*p* value mobility 4–7 versus 0	*p* value mobility 1–3 versu 0
Number of patients receiving IPC at least for 1 day—no. (%)	46 (54.1)	190 (53.4)	677 (53.4)	0.90	0.98
Use of graduated compression stockings—no. (%)	1 (1.2)	3 (0.8)	9 (0.7)	0.48^	0.73^
Pharmacologic thromboprophylaxis					
Prophylactic UFH	49 (57.6)	176 (49.4)	819 (64.6)	0.19	< 0.0001
Prophylactic LMWH	39 (45.9)	205 (57.6)	556 (43.9)	0.72	< 0.0001
Anti-platelet therapy—no. (%)					
Aspirin	20 (23.5)	69 (19.4)	397 (31.3)	0.13	< 0.0001
Clopidogrel	4 (4.7)	27 (7.6)	155 (12.2)	0.04	0.01
New renal replacement therapy—no. (%)	6 (7.1)	24 (6.7)	196 (15.5)	0.04	< 0.0001
Central venous catheters including dialysis catheters—no. (%)					
Femoral	8 (9.4)	51 (14.3)	283 (22.3)	0.0050	0.0010
Jugular or subclavian	37 (43.5)	111 (31.2)	767 (60.5)	0.0020	< 0.0001
Peripherally inserted central catheter	11 (12.9)	32 (9.0)	144 (11.4)	0.66	0.20
None	35 (41.2)	196 (55.1)	345 (27.2)	0.0056	< 0.0001

Continuous variables were not normally distributed and were compared using Mann–Whitney *U* test. Categorical variables were compared using the chi-square test or ^Fisher’s exact test

IPC, intermittent pneumatic compression; UFH, unfractionated heparin; LMWH, low molecular weight heparin; PE, pulmonary embolism

### Outcomes

#### Incident deep-vein thrombosis

**Fig. 2:**
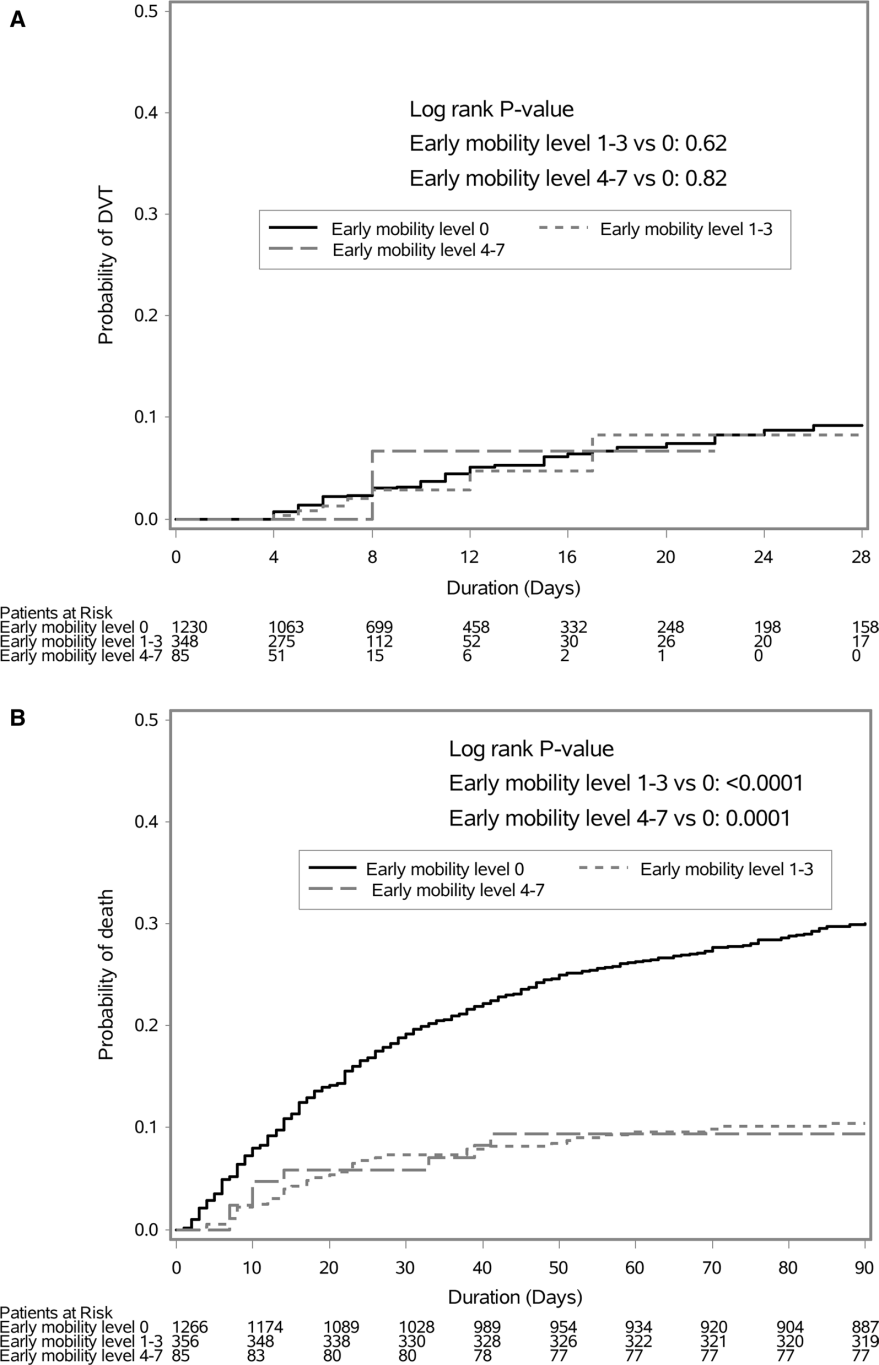
Kaplan–Meier curve for lower-limb deep-vein thrombosis (**A**) and for mortality (**B**) among the three early mobility groups: 4–7, 1–3, and 0. The probability of deep-vein thrombosis was similar in early mobility 4–7 group versus mobility 0 group (*p* = 0.82) and in early mobility 1–3 group versus mobility 0 group (*p* = 0.62). The probability of 90-day mortality was lower in early mobility 4–7 group versus mobility 0 group (*p* = 0.0001) and in early mobility 1–3 group versus mobility 0 group (*p* < 0.0001)

Incident proximal lower-limb deep-vein thrombosis occurred in only 1/58 patient in the early mobility level 4–7 (1.2%; 95% CI 0.0, 3.5%), 7/348 patients in the early mobility group 1–3 (2.0%; 95% CI 0.5, 3.5%) and 50/1230 patients in the early mobility group 0 (4.1%; 95% CI, 3.0–5.2%) (Table 3). The Kaplan–Meier curve (Fig. 2A) shows that the probability of deep-vein thrombosis was similar in early mobility 4–7 group versus mobility 0 group (*p* = 0.82) and in early mobility 1–3 group versus mobility 0 group (*p* = 0.62). On Cox proportional models, early mobility groups 4–7 and 1–3 compared with early mobility group 0 were associated with similar risk of incident lower-limb deep-vein thrombosis (aHR1.19, 95% CI 0.16, 8.90; *p* = 0.87 and 0.91, 95% CI 0.39, 2.12; *p* = 0.83, respectively).

**Table 3:** Outcomes of patients with early mobility levels 4–7, 1–3, and 0

Outcome	Early mobility 4–7 *N* = 85	Early mobility 1–3 *N* = 356	Early mobility 0 *N* = 1267	Mobility 4–7 versus 0		Mobility 1–3 versus 0	
				Hazard ratio (95% CI)	*p* value	Hazard ratio (95% CI)	*p* value
*Primary outcomes*							
Incident proximal lower-limb deep-vein thrombosis—no. (%)	1/85 (1.2)	7/348 (2.0)	50/1230 (4.1)	1.19 (0.16, 8.90)	0.87	0.91 (0.39, 2.12)	0.83
90-day mortality—no. (%)	8/85 (9.4)	37/356 (10.4)	380/1266 (30.0)	0.35 (0.17, 0.70)	0.003	0.39 (0.27, 0.55)	< 0.0001
*Secondary outcomes*							
Prevalent proximal lower-limb deep-vein thrombosis—no. (%)	0/85 (0)	8/356 (2.2)	37/1267 (2.9)	–	–	1.17 (0.53, 2.61)	0.69
Pulmonary embolism—no. (%)	1/85 (1.2)	0/356 (0)	14/1267 (1.1)	1.67 (0.21, 13.57)	0.63	–	0.99
ICU mortality—no. (%)	2/85 (2.4)	18/ 356(5.1)	236/1266 (18.6)	0.53 (0.13, 2.19)	0.38	0.59 (0.36, 0.97)	0.037
28-day mortality—no. (%)	5/85 (5.9)	26/ 356(7.3)	231/1266 (18.2)	0.37 (0.15, 0.90)	0.028	0.48 (0.31, 0.73)	0.0006
Hospital mortality—no. (%)	8/85 (9.4)	32/356 (9.0)	390/1266 (30.8)	0.70 (0.34, 1.42)	0.32	0.49 (0.34, 0.70)	0.0001
				Beta estimate* (95% CI)		Beta estimate* (95% CI)	
Duration of mechanical ventilation—median (IQR)—days	0 (0, 3)	0 (0, 5)	5 (1, 11)	− 0.90 (− 1.24, − 0.57)	< 0.0001	− 0.42 (− 0.59, − 0.25)	< 0.0001
Mechanical ventilation-free days—median (IQR)	28 (25, 28)	28 (22, 28)	21 (2, 26)	0.27 (0.01, 0.52)	0.04	0.19 (0.05, 0.33)	0.009
Duration of vasopressor use—median (IQR)—days	0 (0, 0)	0 (0, 0)	1 (0, 3)	− 1.08 (− 1.51, − 0.64)	< 0.0001	− 0.60 (− 0.81, − 0.39)	< 0.0001
Vasopressor-free days—median IQR)	28 (27, 28)	28 (27, 28)	27 (21, 28)	0.15 (− 0.06, 0.37)	0.17	0.10 (− 0.02, 0.22)	0.09
ICU length of stay—median (IQR)—days	4 (3, 7)	6 (4, 9)	9 (5, 18)	− 0.78 (− 1.00, − 0.55)	< 0.0001	− 0.37 (− 0.49, − 0.25)	< 0.0001
ICU-free days, median (IQR)	24 (21, 25)	22 (18, 24)	16 (0, 22)	0.40 (0.10, 0.71)	0.01	0.27 (0.10, 0.44)	0.002
Hospital length of stay—median (IQR)—days	10 (7, 18)	13 (8, 23)	22 (12, 46)	− 0.66 (− 0.90, − 0.42)	< 0.0001	− 0.33 (− 0.46, − 0.20)	< 0.0001

CI, confidence interval; ICU, intensive care unit; IQR, interquartile range

*Cox regression analysis (for categorical outcomes) and generalized linear mixed model (for continuous outcomes) were used with adjustment for the treatment group, type of heparin, femoral central venous catheters, mechanical ventilation, vasopressor, APACHE II score, and body mass index.

#### Mortality

Among included patients, the 90-day mortality was 8/85 (9.4%) patients in the early mobility level 4–7, 37/356 (10.4%) patients in the early mobility level 1–3 and 380/1266 (30.0%) patients in the early mobility level 0 (*p* = 0.003 for early mobility level 4–7 versus 0 and < 0.0001 for early mobility 1–3 versus 0 (Table 3). The Kaplan–Meier curve (Fig. 2B) shows that survival was significantly higher in the higher early mobility level groups (4–7 and 1–3) compared with group level 0. On Cox proportional analysis, early mobility groups 4–7 and 1–3 compared with mobility group 0 were associated with lower 90-day mortality (aHR 0.47, 95% CI 0.22, 1.01; *p* = 0.052, and 0.43, 95% 0.30, 0.62; *p* < 0.0001, respectively).

#### Secondary outcomes

Pulmonary embolism was diagnosed in 1/85 (1.2%) patient with early mobility level 4–7, 0/356 (0%) patient with early mobility 1–3 and 14/1267 (1.1%) patients with mobility 0 (*p* = 0.63 for early mobility 4–7 versus 0 and *p* = 0.99 for early mobility 1–3 versus 0). Patients with higher early mobility levels (4–7 and 1–3) had significantly more ventilator-free days (median 28 days (IQR 25, 28 days) in the mobility level 4–7 group and 28 days (IQR 22, 28 days) in the 1–3 group versus 21 days (2, 26 days) in the 0 group (adjusted beta coefficient 0.27, 95% CI 0.01, 0.52; *p* = 0.04, and 0.19, 95% CI 0.05, 0.33; *p* = 0.009, respectively) and ICU-free days (median 24 days (IQR 21, 25 days) in the mobility level 4–7 group and 22 days (IQR 18, 24 days) in 1–3 group versus 16 days (IQR 0, 22 days) in 0 group (adjusted beta coefficient 0.40, 95% CI 0.10, 0.71; *p* = 0.01 and 0.27, 95% 0.10, 0.44; *p* = 0.002, respectively). However, there were no significant differences in vasopressor-free days between the mobility level 4–7 group versus 0 and between the mobility level 1–3 group versus 0. The other secondary outcomes of this study are presented in Table 3.

## Discussion

In this cohort of critically ill patients who were expected to stay in the ICU for > 72 h, a minority of patients received high early mobility levels in the first 3 days. Additionally, mobility remained limited throughout the ICU stay. Higher mobility was associated with lower mortality rates, and lower duration of organ support, but with similar incident proximal lower-limb deep-vein thrombosis and pulmonary embolism.

Critical illness is associated with immobility, often confounded by pre-ICU underlying limitation to mobility, cardiopulmonary instability and ICU therapies such as sedation and neuromuscular blockers. In the current study, we found that mobility more than range of motion was provided to only 441/1708 (25.8%) patients during the first 3 days of ICU stay. This finding is not uncommonly observed in ICUs [21, 22]. A point prevalence study of patients with acute respiratory failure in 42 ICUs at 17 US hospitals found that mobility (range of motion and above) from any healthcare provider was provided on 501/770 (65%) patient-days and by physical or occupational therapists on 247/770 (32%) patient-days [21]. Mobility was performed less for mechanically ventilated patients (48% versus 26% for other patients, *p* < 0.001) [21].

Several studies have shown that mobility in critically ill patients was associated with better clinical outcomes, including lower rates of deep-vein thrombosis, ventilator-associated pneumonia, and pressure injury, increased muscle strength and shorter duration of mechanical ventilation, and ICU and hospital stay [18, 23, 24]. In a pre-post quasi-experimental study in trauma patients (1,044 patients in the pre- and 1,132 patients in the post-intervention cohort), an early mobility program was associated with lower rate of deep-vein thrombosis (6.7% versus 10.9%, *p* < 0.01; adjusted relative risk 0.67, 95% CI 0.50, 0.90) [25]. Another pre-post quasi-experimental study evaluated a structured progressive mobility protocol in trauma and burns patients (184 patients in the pre- and 159 patients in post-intervention cohort) observed lower rate VTE in postintervention cohort (7.5% versus 21%, *p* = 0.0004) [14]. A systemic review and meta-analysis also showed that mobility reduced the risk of deep-vein thrombosis (risk ratio 0.16; 95% CI 0.06, 0.47) [18]. This was based on 7 randomized controlled trials, mostly with small sample sizes (total of 730 patients) and significant biases [18]. Additionally, the studies used different definitions of mobility [17, 18]. In the current study, we found that higher early mobility levels were associated with similar incident lower-limb deep-vein thrombosis and pulmonary embolism compared with lower mobility level (range of motion) in patients expected to stay > 72 h in the ICU. As all study patients were on pharmacologic prophylaxis by study design, it is possible that early mobility has little impact on VTE prevention in such a setting, especially that mobilization was probably relatively short in duration for each session relative to the rest of the time during the day spent in the ICU. We also found that higher mobility levels were associated with lower mortality and less organ support. A meta-analysis of randomized and controlled trials found that mobility had no impact on short- and long-term mortality in ICU patients but led to more days alive and out of hospital to day 180 [24]. More recently, The Treatment of Mechanically Ventilated Adults with Early Activity and Mobilization (TEAM) trial randomized 750 adult patients on mechanical ventilation and found that an increase in early active mobilization (sedation minimization and daily physiotherapy) did not result in a significantly greater number of days alive and out of hospital compared with the usual level of mobilization in the ICU [26]. In this trial, both groups received active physiotherapy; the number of days per patient when physiotherapy assessment occurred was 0.94 ± 0.11 in the early active mobilization group and 0.81 ± 0.24 in the usual care group [26]. The limited separation in treatment exposure between the two groups and the active mobility program in the control arm, which do not reflect mobility practices in most ICUs, keep this question open [21, 27].

The strengths of the study include that data came from a multicenter randomized controlled trial wherein twice-weekly structured surveillance for lower-limb deep-vein thrombosis was performed in a large number of patients, thus reducing ascertainment bias. Our study characterized important associations between higher versus lower early mobility levels and important clinical outcomes besides mortality. VTE is rarely studied as an outcome of mobility. Our review of clinicaltrials.gov database showed that none of the 10 ongoing randomized controlled trials that were evaluating mobility in critically ill patients had VTE as a pre-specified outcome (Additional file 1: Table S5). Our study also has limitations. First, we did not have detailed description of mobility, such as duration, intensity, whether being active or passive, and contraindications, including the practices of sedation and neuromuscular blockade. Second, we could not analyze patients according to individual mobility level, given the low number of patients who had higher mobility levels. Third, although our findings indicated that early mobility was associated with lower mortality in ICU patients, we cannot establish a causal relationship between mobility and mortality. The observed association between early mobility and mortality and the lack of association between early mobility and lower-limb deep-venous thrombosis might be related to differences across patient groups in baseline characteristics including VTE risk factors and care processes, and other unmeasured confounders that were not accounted for in our multivariable analysis models. It is likely that the illness severity of the patients who had a higher mortality rate led them to being less mobile.

## Conclusions

Only a small proportion of critically ill patients had a high level of mobility early in their ICU stay. There was no association between early mobility levels and lower-limb deep-vein thrombosis. Although early mobility level was independently associated with lower mortality risk, this association does not establish causality and randomized controlled trials are required to assess whether and to what extent this association is modifiable.

## Supplementary Information

**Additional file 1.** Supplementary tables.

## Data Availability

The datasets used and/or analyzed during the current study are not publicly available but are available from the corresponding author on reasonable request.
