# Supplementary material for: Association of early mobility with the incidence of deep-vein thrombosis and mortality among critically ill patients: a post hoc analysis of PREVENT trial
Source: Crit Care. 2023 Mar 3;27:83. doi: 10.1186/s13054-023-04333-9 (PMC9985278; doi:10.1186/s13054-023-04333-9)
Supplement: Supplementary file 1 — Additional file 1. Supplementary tables. [file 13054_2023_4333_MOESM1_ESM.docx]

**Supplement to:**

Association of early mobility with the incidence of deep-vein thrombosis and mortality among critically ill patients: a post hoc analysis of PREVENT trial

# Table S1: The Agency for Healthcare Research and Quality definitions of the different mobility levels for mechanically ventilated patients.

| **Mobility level** | **Definition** |
| --- | --- |
| **0** | **Passive range of motion**: passively rolled or exercised by staff, but not actively moving (includes raising head of bed to upright position without patient participation in movement, chest physical therapy, and splinting) |
| **1** | **Transfer from bed to chair without standing**: hoist, passive lift, or slide to the chair without standing |
| **2** | **Sitting in bed/exercises in bed**: any activity in bed, including active rolling, bridging, active exercises, active movement from supine to sitting position, use of cycle ergometer, use of tilt table, not moving out of bed or over the edge of the bed |
| **3** | **Sitting at edge of bed**: actively sitting over the side of the bed with some trunk control (may be assisted) |
| **4** | **Standing**: weight bearing through feet in standing position with or without assistance; may include use of a standing lifter |
| **5** | **Transfer from bed to chair with standing**: able to step or shuffle through standing to chair; this involves actively transferring weight from one leg to another to move to chair |
| **6** | **Marching in place**: able to walk in place by lifting alternate feet (must be able to step at least four times, two for each foot) with or without assistance |
| **7** | **Walking**: walking away from the bed/chair by at least four steps (two for each foot) assisted by a person/people or gait aid, or unassisted |

**Table S2:** Mobility practices in the participating centers at the time of the trial. Sixteen out of 19 centers responded. Sites with <10 patients recruited were not included in the survey.

| **Item** | **Response** |
| --- | --- |
| A protocol for early mobility was implemented in the ICU, n/N (%) | 9/16 (56.3%) |
| A physiotherapy specialist provided mobility to patients in the ICU, n/N (%) | 14/16 (87.5%) |
| Physiotherapy specialist to patient ratio, median (interquartile range) | 1: 10 (10, 12) |
| Number of days physiotherapist provided treatments per week, median (interquartile range) | 5 (5, 5.75) |
| Predefined criteria for eligibility for mobility in the ICU, n/N (%) | Yes 8/16 (50%) |
| Bedside nurses routinely provide range of motion exercises to ICU patients, n/N (%) | Yes 8/16 (50%) |
| Mobility practices were audited in the ICU, n/N (%)? | Yes 2/16 (12.5%) |
| Early mobility was a quality indicator in your ICU, n/N (%) | Yes 5/16 (31.3%) |

**Table S3:** Early mobility level in the study patients according to their country.

|  | **Saudi Arabia**  **(N=1313)** | **India**  **(N=182)** | **Canada**  **(N=147)** | **Australia**  **(N=65)** |
| --- | --- | --- | --- | --- |
|  |  |  |  |  |
| Early mobility level 0 | 1067 (81.3) | 86 (47.3) | 87 (59.2) | 26 (40) |
| Early mobility level 1-3 | 218 (20.4) | 87 (47.8) | 43 (29.3) | 8 (12.3) |
| Early mobility level 4-7 | 28 (2.1) | 9 (4.9) | 17 (11.6) | 31 (47.7) |

**Table S4:** Additional baseline characteristics of the study patients.

|  | **Early Mobility 4-7 N=85** | **Early Mobility 1-3 N=356** | **Early Mobility 0 N=1267** | **P-value mobility 4-7 vs. 0** | **P-value mobility 1-3 vs. 0** |
| --- | --- | --- | --- | --- | --- |
| **Location prior to ICU admission – no. (%)** |  |  |  |  |  |
| Emergency room | 45 (52.9) | 192 (53.9) | 621 (49.0) | 0.22 | 0.28 |
| Hospital ward | 21 (24.7) | 101 (28.4) | 390 (30.8) |  |  |
| Operating room | 14 (16.5) | 31 (8.7) | 134 (10.6) |  |  |
| Other hospital (ICU or ward) | 4 (4.7) | 32 (9.0) | 114 (9.0) |  |  |
| Other | 1 (1.2) | 0 | 8 (0.6) |  |  |
| **Pre-ICU VTE risk factors – no. (%)** |  |  |  |  |  |
| None | 53 (62.4) | 185 (52.0) | 491 (38.8) | <0.0001^ | <0.0001^ |
| Hospitalization in the past 3 months for any reason (excluding this hospital admission) | 15 (17.6) | 60 (16.9) | 291 (23.0) |  |  |
| Paralysis or immobilization of a lower or upper extremity related to stroke or injury prior to this hospital admission | 2 (2.4) | 24 (6.7) | 137 (10.8) |  |  |
| Active malignancy (treatment within past 6 months or palliation) | 5 (5.9) | 30 (8.4) | 127 (10.0) |  |  |
| Recent surgery (in the last 48 hrs) | 12 (14.1) | 26 (7.3) | 123 (9.7) |  |  |
| Acute stroke (this hospital admission) | 0 | 5 (1.4) | 63 (5.0) |  |  |
| Trauma | 4 (4.7) | 30 (8.4) | 111 (8.8) |  |  |
| History of malignancy (past 5 years; other than non-melanoma skin cancer) | 3 (3.5) | 26 (2.1) | 10 (2.8) |  |  |
| Personal history of VTE | 3 (3.5) | 13 (1.0) | 3 (0.8) |  |  |
| Family history of VTE | 1 (1.2) | 3 (0.2) | 2 (0.6) |  |  |
| Known thrombophilic state | 0 | 0 | 0 |  |  |
| Post-partum (within 3 months) | 1 (1.2) | 3 (0.2) | 2 (0.6) |  |  |
| Estrogen therapy | 1 (1.2) | 1 (0.1) | 1 (0.3) |  |  |
| Others |  | 40 (3.2) | 9 (2.5) |  |  |
| **Laboratory results at baseline - mean (SD)** |  |  |  |  |  |
| INR (highest) | 1.2±0.2 | 1.2±0.3 | 1.3±0.7 | 0.68 | 0.31 |
| Creatinine (µmol/L) - median (IQR) | 73.0 (59.0, 109.0) | 92.0 (62.0, 174.0) | 83.5 (61.4, 144.0) | 0.02 | 0.08 |
| Platelets (10^9^/L) | 250.0±113.8 | 246.0±123.3 | 229.4±117.6 | 0.53 | 0.02 |
| PTT (sec) | 32.2±10.7 | 33.3±11.0 | 30.6±11.6 | 0.61 | <0.0001 |
| Hemoglobin (g/L) | 101.2±38.6 | 104.7±96.6 | 110.5±102.9 | 0.11 | 0.26 |

SD: standard deviation; BMI-body mass index; APACHE: Acute Physiology and Chronic Health Evaluation; INR: international normalized ratio; PTT: partial thromboplastin time; VTE: venous thromboembolism; IQR: interquartile range

To convert the values for creatinine to mg/dl, divide by 88.4.

Continuous variables were not normally distributed and were compared using Mann–Whitney U test (two-group comparisons) and Kruskal Wallis test (3 group comparisons).

Categorical variables were compared using the chi-square test or ^Fisher’s exact test.

**Table S5:** Ongoing randomized controlled trials on mobility in adult critically ill patients that are registered in clinicaltrials.gov (Search performed in September 2022).

| **Trial identifier/Principal investigator (country)/** | **Title** | **Status** | **Population** | **Intervention/Comparator** | **Outcome measures** |
| --- | --- | --- | --- | --- | --- |
| ClinicalTrials.gov: NCT04582760  Gee Young Suh (Korea) | Early mobilization in Ventilated sEpsis & acute Respiratory failure Study: *EVER Study* | Multicenter  Active, recruiting | 200 patients on mechanical ventilation > 48 hrs for acute respiratory failure and sepsis | Intervention:  mobilization program administered for 30 mins per session, two sessions per day, 7 days per week  Control: bedside physical therapy administered for 30 mins per session, one session per day, 5 days per week | Primary: Functional Status Score for the Intensive Care Unit  Secondary: ICU/Hospital stay, Duration of MV, Delirium, MRC score, hand grip.  VTE was not a prespecified outcome. |
| ClinicalTrials.gov: NCT03406494  Yan Zhang (China) | Efficacy and Safety of a Multicomponent Physical Therapy Program in Mechanically Ventilated Patient With Sepsis *(PTMVP)* | Multicenter,  Unknown | 800 patients on mechanical ventilation > 48 hrs for acute respiratory failure and sepsis | Intervention: Early multicomponent physical therapy program plus sepsis standard therapy  Control: sepsis standard therapy | Primary: ICU 28-day mortality  Secondary: ICU/Hospital stay, duration of MV, delirium, health-related quality of life/MRC score, diaphragmatic dysfunction.  VTE was not a prespecified outcome. |
| ClinicalTrials.gov: NCT02872792  Gaétan Beduneau (France) | Early Mobilisation in Intensive Care Unit: Interest of Cyclo-ergometry in Patients With Septic Chock (MUEVELO) | Terminated | 122 enrolled out of planned 234 patients with septic shock diagnosed > 24 hours before enrolment, on mechanical ventilation | Intervention: early mobilization with cyclo-ergometer  Control: standard physiotherapy | Primary: Number of days between hemodynamic stability and ICU discharge  Secondary: Number of days between hemodynamic stability and the removal of sedation, duration of MV  VTE was not a prespecified outcome. |
| ClinicalTrials.gov: NCT05118529  Annika Söderberg (Sweden) | Comparison of Physiological Response and Experience Between Sitting in Bed and Sitting in a Chair in Patients in Intensive Care | Active, rectruiting | 30 patients admitted to the ICU | Intervention: Sitting in chair during 20 minutes  Control: sitting in bed bed with 60 degrees elevated back-rest during 20 minutes | Primary: Arterial oxygen pressure, Arterial carbon dioxide pressure  Secondary: Blood pressure, SpO2, Perceived pain, Perceived exertion, Perceived satisfaction  VTE was not a prespecified outcome. |
| ClinicalTrials.gov Identifier: NCT05401461  David McWilliams (United Kingdon) | Does Mobilisation in the Evening Reduce the Incidence of Delirium in Patients Admitted to Intensive Care: a Mixed-methods, Randomised Controlled Feasibility Study | Not yet recruiting | 60 patients admitted to the ICU | Intervention: Evening mobilisation delivered between 7pm and 9pm  Control: standard care which incorporates physiotherapy and mobilisation as appropriate between 8am and 5pm | Primary: Recruitment rate Proportion of patients agreeing to take part out of all those invited, Retention rate, Proportion of participants who complete the intervention, Intervention fidelity  Secondary: Incidence of delirium, Duration of delirium, Sleep quality, Mobility level at ICU discharge  VTE was not a prespecified outcome. |
| ClinicalTrials.gov: NCT05038930  Kirsten Møller (Denmark) | Mobilising Patients With Severe Brain Injury in Intensive Care (MAWERIC) | Recruiting | 22 patients with severe brain injury | Intervention: progressive mobilization using the Sara Combilizer®  Control: sedentary protocol will follow the same four phases as the intervention protocol only the patient will remain in the supine position | Primary: Change in partial oxygenation of brain tissue  Secondary: change in hemodynamics, intracranial pressure and other indicators  VTE was not a prespecified outcome. |
| ClinicalTrials.gov: NCT03771014  Rebecca Cusack (United Kingdom) | EMPRESS: A Feasibility Study of early Mobilization Programmes in Critical Care | Recruiting | 90 patients > 42 yr-old admitted to the ICU | Intervention: Patients will receive standard physiotherapy regimen plus 2 x 30-minute rehabilitation sessions 5 days per week.  Control: standard physiotherapy regimen | Primary: Physical Function ICU Test-score  Secondary: Medical Research Council Manual Muscle Test Sum Score, Hand-held dynamometry, Chelsea Critical Care Physical Assessment tool, ICU Mobility Scale, Clinical Frailty Score, Barthel Index for Activities of Daily Living, Six minute walk test, The Hospital Anxiety and Depression Scale, WHO Disability Assessment Schedule 2.0, EQ-5D-5L  VTE was not a prespecified outcome. |
| ClinicalTrials.gov Identifier: NCT03554811  Paul D Smith  (United States) | Early Rehabilitation Using Functional Electrical Stimulation Assisted Supine Cycling in the ICU | Recruiting | 32 patients expected to stay in the ICU for > 4 days | Intervention: functional electrical stimulation assisted supine cycling (FESC) within 48 hours of ICU admission and will undergo up to 1 hour of supine cycling daily, 5 days per week for 28 days, or until discharge from ICU  Control: standard ICU exercise and mobility interventions | Primary: Percent change of rectus femoris cross-sectional area  Secondary: Diaphragm muscle thickness, Muscle strength Muscle strength, Physical function, Quality of life, Cognition, Hospital length of stay, ICU length of stay, Duration of mechanical ventilation |
| ClinicalTrials.gov Identifier: NCT05279547  Daniel Langer (Belgium) | Locomotor Muscle Oxygenation and Activation During Acute Interval Compared to Constant-load Bed-cycling Exercise | Not yet recruiting | 100 patients with MV for > 48 hours and expected to remain in the ICU for more than an additional 48 | Intervention: First constant-load then interval bed-cycling protocol  Control: First interval then constant-load bed-cycling protocol) | Primary: Differences between bed-cycling protocols in fractional oxygen saturation for each measured region of the m. quadriceps femoris, Differences between bed-cycling protocols in activation, and hemodynamic events.  Secondary: Differences in Relative dispersion of fractional oxygen saturation among the different regions of quadriceps femoris, Differences between exercise protocols in Relative dispersion of fractional oxygen saturation among the different regions of quadriceps femoris, Differences between exercise protocols in oxygenated hemoglobin/myoglobin, Differences between bed-cycling protocols in heart rate, mean arterial blood pressure, respiratory rate, minute ventilation  VTE was not a prespecified outcome. |
| ClinicalTrials.gov Identifier: NCT05450120  Jacqueline Vianna (Brazil) | Neuromuscular Electrical Stimulation and Functional Rehabilitation Protocol in Critically Ill Patients With SARS-CoV-2 Variants in ICUs With Limited Resources: A Randomized Clinical Trial | Active, not recruiting | 88 patients admitted in ICU for >72 hrs, on MV for > 48hrs, with acute respiratory distress syndrome secondary to COVID-19, with shock or organ failure | Intervention: Functional rehabilitation protocol associated with neuromuscular electrical stimulation  Control: Functional rehabilitation protocol | Primary: Skeletal muscle strength, Functional independence  Secondary: Mechanical ventilation, Survival  VTE was not a prespecified outcome. |

**The Saudi Critical Care Trials Group**

| **Management Committee** | Dr. Yaseen M. **Arabi**  Dr. Abdulaziz **Al-Dawood**  Dr. Sami J. **Alsolamy**  Sheryl Ann I. **Abdukahil**  Lara Y **Afesh**  Jesna **Jose** |
| --- | --- |
| **Writing Committee** | Dr. Yaseen M. **Arabi**  Dr. Hasan **Al-Dorzi**  Sheryl Ann I. **Abdukahil**  Jesna **Jose**  Dr. Karen EA **Burns**  Dr. Sangeeta **Mehta**  Prof. Simon **Finfer** |
| **Data Monitoring Committee** | Prof. Kathryn **Rowan** (Chair)  Dr. Lehana **Thabane**  Dr. David **Garcia** |

| **Collaborators (*Sites are in alphabetical order)*** | |
| --- | --- |
| **Saudi Arabia** | |
| **Asir Central Hospital, Abha** | Dr. Ali **Al Bshabshe**  Dr. Abdulmoniem **Albahar**  Dr. Ali **Alamri**  Bincy **Mathew**  Nora **Assiri**  Dr. Ausama Omar **Ismaeil** |
| **King Abdulaziz Hospital, Ahsa** | Dr. Abdulsalam **Al Aithan**  Shahinaz **Bashir**  Dr. Syed **Hassan**  Jecel **Natavio**  Priscilla **Guiang**  Minda **Baisas** |
| **King Abdulaziz Medical City, Jeddah** | Dr. Fahad **Al-Hameed**  Dr. Gulam **Rasool**  Dr. Jalal **Rifai**  Ali S. **Mohamed**  Ohoud **Al Orabi**  Ferdos **Alahmary** |
| **King Abdulaziz Medical City, Riyadh** | Dr. Yaseen M. **Arabi**  Dr. Abdulaziz **Al-Dawood**  Dr. Sami J. **Alsolamy**  Dr. Mohamed **Hegazy**  Dr. Maamoun **Dbsawy**  Sheryl Ann I. **Abdukahil**  Lara Y **Afesh**  Jesna **Jose**  Dr. Ghassan **Al-Maziad**  Dr. Musharaf **Sadat**  Eman **Al Qasim**  Ahmad **Deeb**  Muhammed **Rafique**  Aron **Toledo**  Felwa **bin Humaid**  Amjad **Alaskar**  Ahmad **Al Khalaf**  Ahmed **Kanfer**  Albatole **Gorban**  Helen **Batin**  Mohammed **Al Shehri** |
| **King Fahad Medical City** | Dr. Mohammed **Almaani**  Dr. Hani **Lababidi**  Dr. Husain **Abdulmuthalib**  Pendo **Ntinika**  Emelinda **Ramos**  Ibrahim **AlEidan**  Nona **Bacani**  Dr. Ahmad **Al Jefri** |
| **King Fahd Hospital of the University, Imam Abdulrahman Bin Faisal University, Dammam** | Dr. Mohammed S **Alshahrani**  Laila Perlas **Asonto**  Kathrina **Libunao-de Loyola**  Charlene **Mapusao**  Dr. Mohammed **ElGalhoumy**  Dr. Ahmed Ali Abdl-Hali **Hassan**  Esperanza **Dipasupil**  Norma **Toledo** |
| **King Faisal Specialist Hospital and Research Center, Jeddah** | Dr. Imran **Khalid**  Dr. Ismael **Qushmaq**  Maryam **Imran**  Manahil **Imran**  Lenith **Salazar**  Nouf **Sulimani**  Jessel **Teves**  Johanna **Vega**  Joan **Ferrer** |
| **King Faisal Specialist Hospital and Research Center, Riyadh** | Dr. Hassan **Hawa**  Dr. Khalid **Maghrabi**  Dr. Mohammad **Hijazi**  Dr. Musaab **Abdelhai**  Ellen Joy **Pagunsan**  Marketa **Vinklerova**  Dr. Salahudin **Elnaas**  Muna **Al Zahrani**  Manal **Mustafa**  Lamya **Al AbdulAziz**  Nouf **Al Amri** |
| **Prince Sultan Military Medical City** | Dr. Ghaleb A. **Almekhlafi**  Dr. Yasser **Mandourah**  Dr. Sahar **Hassan**  Dr. Emad **Al Amodi**  Dr. Osama **Elfaki**  Dr. Ahmad **Alenazy**  Dr. Dina Al **Sufiani**  Dr. Bander **Alanazi**  Kholoud **Alharbi**  Shatha **Awad**  Ma. Raylin Cubio **Cabal**  Jean S. **Valerio**  Dr. Mohammed **Alkhader**  Esperanza **Bautista** |
| **Canada** | |
| **Mount Sinai Hospital** | Dr. Sangeeta **Mehta**  Sumesh **Shah**  Erik **Tamberg**  Crystal **Angaran**  Carlos **Arrazola**  Ashley **Briggs**  Angela **Calabrese**  Lyndsey **Cameron**  Erin **Cham**  Doreen **Chu**  Saerom **Chung**  Krista **Colley**  Ann **Downey**  Amy **He**  Kelli **Jackson**  Heather **Macdonald**  Oleksander **Malyarchuk**  Mirela **Papadhima**  Jamie **Song**  Anjuli **Sookoo**  Melissa **Wong**  Qiang **Zhang**  Tingting **Zhang**  Michelle **Zhen**  Ning **Zhu** |
| **Saint Boniface General Hospital** | Dr. Ryan **Zarychanski**  Nicole **Marten**  Dr. Justin **Lys** |
| **Saint Michael’s Hospital** | Dr. Karen EA **Burns**  Dr. Jan **Friedrich**  Dr. Andrew **Baker**  Orla **Smith**  Marlene **Santos**  Gyan **Sandhu**  Jennifer **Hodder**  Imrana **Khalid**  Dr. Paraskevi **Vlachou**  Dr. Anish **Kirpalani**  Dr. Errol **Colak**  Dr. Gevork **Mnatzakanian**  Carolyn **Trottier**  Junwu **Mu**  Roya **Akbari**  Meng **Qin**  Siu Chu **Lam**  Alexandra **Karolczyk**  Carmen **Lau**  Jessica **Fu**  Cara **Leis**  Glenn **Gabrielpillai**  Bao Truong (Leo) **Tran**  Chrystal **Douglas**  Iris **Fok**  Jieqi **Ma**  Victoria **Babysheva**  Lin **Lan** |
| **Sunnybrook Health Sciences Centre** | Dr. Robert **Fowler**  Dr. Neill **Adhikari**  Dr. Damon **Scales**  Nicole **Marinoff**  Adic **Perez**  Navjot **Kaur**  Vivekanandan **Thayalasuthan**  Sonia **Welsh** |
| **University of Alberta Hospital** | Dr. Michael **Jacka**  Dr. Sean **Bagshaw**  Nadia **Baig**  Lorena **McCoshen**  Dr. Gavin **Low**  Marina **McToal** |
| **Australia** | |
| **Gosford Hospital** | Dr. Atul **Gaur**  Katrina **Ellis**  Mary **White**  Dr. Rajiv **Rattan** |
| **Royal North Shore Hospital** | Prof. Simon **Finfer**  Associate Professor Anthony **Delaney**  Elizabeth **Yarad**  Anne **O’Connor**  Frances **Bass**  Naomi **Hammond**  Julia **Pilowsky**  Sharon **Mar**  Melissa **Owen**  Simon **Bird**  Dr. Charles **Fisher**  Linda **Thebridge**  Louise **McIntosh**  Zoe **Li**  David **Su**  Katherine **Prasad** |
| **St Vincent’s Hospital, Sydney** | Dr. Hergen **Buscher**  Claire **Reynolds**  Nerilee **Baker**  Karlee **McCann**  Elizabeth **Pluis** |
| **India** | |
| **King George’s Medical University** | Dr. Zia **Arshad**  Sachin Kumar **Srivastava**  Avinash **Singh**  Archit **Deva**  Dr. Shailesh Kumar **Singh**  Dr. Amit **Kushwaha** |
| **Medanta, The Medicity** | Dr. Yatin **Mehta**  Joby V. **George**  Dr. Chitra **Mehta**  Dr. Ashish **Kumar**  Jyoti **Chandel**  Dr. Gaurav **Kochhar**  Bhuwan **Singh** |
| **Vijaya Orthopedic and Trauma Center** | Dr. Mrityunjaya **Uppin**  Devesh **Kumar**  Anit **Singh**  Dr. Vinayak **Kabate** |
